# Production of functional eggs and sperm from in vitro-expanded type A spermatogonia in rainbow trout

**DOI:** 10.1038/s42003-020-1025-y

**Published:** 2020-06-15

**Authors:** Yoshiko Iwasaki-Takahashi, Shinya Shikina, Masaya Watanabe, Akira Banba, Masaru Yagisawa, Kasumi Takahashi, Ryo Fujihara, Takafumi Okabe, Delgado M. Valdez Jr, Akihiro Yamauchi, Goro Yoshizaki

**Affiliations:** 1 Department of Marine Biosciences, Tokyo University of Marine Science and Technology, Tokyo, 108-8477 Japan; 2 Institute of Marine Environment and Ecology, National Taiwan Ocean University, Keelung, Taiwan

**Keywords:** Transdifferentiation, Spermatogenesis

## Abstract

Combining cryopreservation of germline stem cells (GSCs) with their subsequent transplantation into recipient fish is a powerful tool for long-term preservation of genetic resources of endangered fishes. However, application of this technique has been limited because endangered species sometimes have small gonads and do not supply enough GSCs to be used for transplantation. This limitation could be overcome by expanding GSCs in vitro, though this has been difficult due to the complexity of reconstructing the gonadal microenvironment that surrounds GSCs. Here, we describe a novel method of in vitro expansion of rainbow trout GSCs using a feeder layer derived from Sertoli cells and a culture medium containing trout plasma. A transplantation assay demonstrated that the in vitro-expanded GSCs exhibited stem cell activity and potency to produce functional eggs, sperm, and eventually healthy offspring. In vitro expansion of GSCs can aid in rescuing fishes that are on the verge of extinction.

## Abstract (web summary)

Yoshiko Iwasaki-Takahashi et al. report the production of fertilization-capable eggs and sperm from rainbow trout spermatagonia, for application to endangered species preservation. Their method avoids the need for limited numbers of germline stem cells for transplantation, instead allowing in vitro expansion of these cells using a Sertoli cell-derived feeder layer.

## Introduction

Many fish species are experiencing rapid population declines, with several species facing extinction^1,2^. Therefore, long-term preservation of fish genetic resources is increasingly important for the conservation of endangered fish species. Due to the lack of an available method to cryopreserve fish eggs or embryos^3^, a combination of cryopreservation of germline stem cells (GSCs) and their subsequent transplantation into recipient fish is a practical alternative for long-term preservation of genetic resources of endangered fish species or strains^4,5^. However, this method suffers from difficulties, including that the number of GSCs harvested from donor individuals are not sufficient for transplantation. This is a critical limitation for species with small testes or ovaries. This limitation could be overcome by using in vitro-expanded GSCs as donor cells for transplantation.

Although several attempts have been made in various fish species^6-10^, there have been no reports demonstrating the production of functional eggs derived from in vitro-cultured GSCs. We previously found that when GSCs harvested from the testes of rainbow trout were transplanted into female recipients, they were incorporated into recipient ovaries and eventually differentiated into fully functional eggs with full developmental potency^11^. Further, the available number of GSCs is more abundant in testes than in ovaries^11,12^. Therefore, the current study aimed to develop an in vitro culture system using testicular, not ovarian, germ cells in order to obtain a substantial number of GSCs that can produce functional eggs after transplantation into recipients.

One promising strategy to establish a GSC culture system is to reconstruct the cellular microenvironment of the gonad in vitro. Teleosts, including rainbow trout, have a cystic testis structure, in which developing germ cell syncytia are individually surrounded by Sertoli cells^13^. It is noteworthy that germ cells are encapsulated by Sertoli cells from the single type A spermatogonium (ASG) stage^13^, which should include spermatogonial stem cells. Therefore, we predicted that Sertoli cells has an indispensable role in creating a suitable microenvironment to support spermatogonial stem cells in fish testes. Further, fish Sertoli cells are mitotically active and continue to proliferate in adult testes^14^. Therefore, we produced a Sertoli cell line to use as a feeder layer for spermatogonial stem cell expansion in vitro in rainbow trout in order to partially reconstruct the cellular microenvironment of rainbow trout testes.

In the present study, Sertoli cells were first isolated from juvenile rainbow trout with singly-isolated ASG and cultured in vitro to produce a feeder layer. Second, we developed a method for the in vitro expansion of rainbow trout ASGs using the derived feeder layer and a newly developed culture medium containing rainbow trout blood plasma. Furthermore, the in vitro-expanded ASGs were confirmed to exhibit stem cell activity and possessed potency to differentiate into functional eggs and sperm in recipient gonads, allowing for production of viable offspring following fertilization.

## Results

### In vitro culture of Sertoli cells

**Fig. 1:**
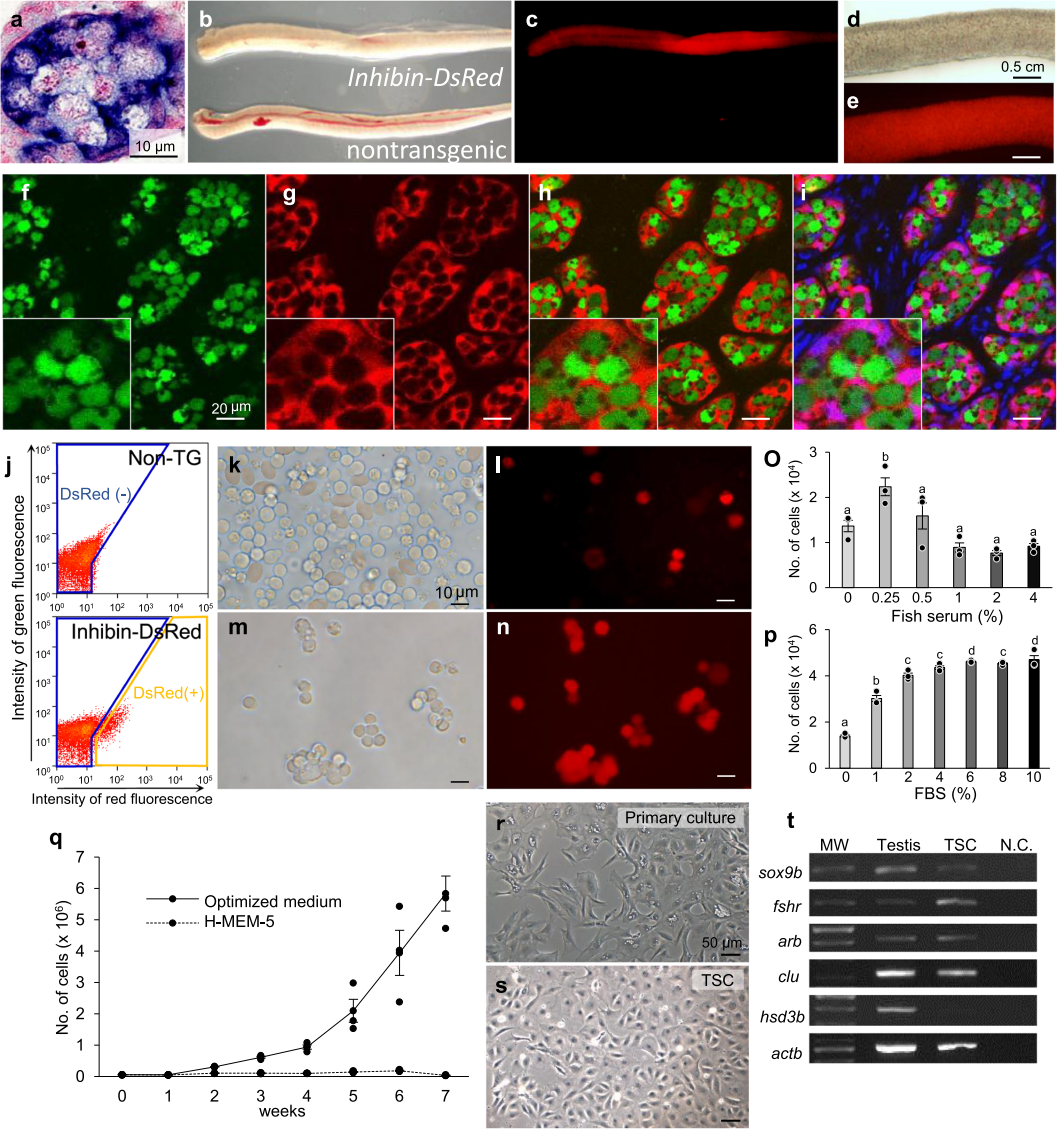
Establishing the *inhibin*-*DsRed* transgenic rainbow trout strain and Sertoli cell line. **a** Localization of *inhibin α* mRNA in immature testes assessed by in situ hybridization. *Inhibin* mRNA was expressed in Sertoli cells surrounding ASGs. **b** Micrograph of immature testes from 13-month-old *inhibin*-*DsRed* transgenic and nontransgenic rainbow trout. **c** Fluorescent view of the same field as **b**. **d**, **e** Higher magnification views of an immature testis from an *inhibin*-*DsRed* transgenic rainbow trout. **f**–**i** Confocal microscopy of an immature testis from a 13-month-old *vasa*-*gfp*/*inhibin*-*DsRed* double transgenic rainbow trout. ASGs (green) were surrounded by Sertoli cells (red). Cell nuclei were stained with Hoechst 33342 (blue). Insets show a higher magnification of each photo. **j** Flow cytometry analysis of immature testes from nontransgenic (Non-TG) and *inhibin*-*DsRed* transgenic rainbow trout. The gated regions with blue lines and orange lines indicate the DsRed(−) population and DsRed(+) population, respectively. **k**–**n** Bright and U-MWIG2 filter-fluorescent views of the dissociated immature testicular cells (**k**, **l**) and Sertoli cells isolated by flow cytometry (**m**, **n**) from *inhibin*-*DsRed* transgenic rainbow trout. **o** The effect of fish serum on TSC growth. **p** The effect of FBS on TSC growth. The Sertoli cell counts were determined following 14 days of culture. Data are shown as the mean ± SEM (*n* = 3). Groups with different letters are significantly different based on one-way ANOVA followed by Tukey’s multiple comparisons test (*P* < 0.05). **q** Comparison of Sertoli cell counts under the optimized medium (ERDF medium supplemented with 6% FBS, and 0.25% fish serum) and a standard culture medium for salmonids (Hank’s MEM supplemented with 5% FBS) over a 7-week culture period. Data are shown as the mean ± SEM (*n* = 3). **r** Micrograph of isolated Sertoli cells following 26 days in primary culture. **s** Representative micrograph of the TSC line. **t** Expression of *sox9b*, *fshr*, *arβ*, and *clu* in the established TSC line assessed by RT-PCR analysis. *actb* and *hsd3b* were used as an internal control and a negative control, respectively, for RT-PCR analysis. cDNA from an immature testis (Testis) was used as the positive control. Reactions lacking the template (N.C.) were included as the negative control.

To establish a culture condition that allowed for expansion of trout ASGs in vitro, we first attempted to establish a feeder cell for ASGs. In fish testes, ASGs are encapsulated by Sertoli cells that regulate the survival, proliferation, and differentiation of male germ cells^13^. The *inhibin* α gene is specifically expressed in Sertoli cells in trout testes (Fig. 1a). Therefore, we produced a transgenic rainbow trout strain, *inhibin*-*DsRed* transgenic trout, which carries the *DsRed* gene under control of the *inhibin* α promoter to isolate Sertoli cells and use them as a feeder for the trout ASG culture. Microscopically, immature testes of the *inhibin*-*DsRed* transgenic strain exhibited strong red fluorescence (Fig. 1b–e). Confocal microscopic analysis of *vasa*-*gfp*^15,16^/*inhibin*-*DsRed* double transgenic rainbow trout further confirmed that Sertoli cells were specifically labeled with DsRed (Fig. 1f–i). Next, we isolated DsRed-labeled Sertoli cells by combining enzymatic dissociation of testes with flow cytometry (Fig. 1j–n). The isolated Sertoli cells tended to extend on the culture plate soon after seeding, indicating that the Sertoli cells were isolated in a viable state. Subsequently, we developed a culture condition to support the in vitro expansion of Sertoli cells by optimizing the fish serum (Fig. 1o) and fetal bovine serum (FBS) concentrations (Fig. 1p) in the culture medium. Under the optimized culture medium (ERDF medium supplemented with 10 mM HEPES, 0.25% fish serum, and 6% FBS), Sertoli cells showed stable proliferation over the 7-week test period (Fig. 1q). By contrast, Sertoli cell proliferation was not observed in a standard culture medium for salmonid cells (Hank’s MEM supplemented with 25 mM HEPES and 5% FBS; H-MEM-5; Fig. 1q). Sertoli cells were expanded in culture for more than 1 year with multiple passages (>48 times) in the optimized medium. At this point, we recognized the cells as a constituted cell line, called the trout Sertoli cell (TSC) line. Morphologically, TSCs resembled epithelial cells and retained a close resemblance to those in the primary culture (Fig. 1r, s). RT-PCR analysis showed that the TSC line expressed a subset of typical Sertoli cell markers (*sox9b*, *fshr, arβ*, and *clu*) but not a Leydig cell marker (*hsd*3*β*) (Fig. 1t). Unexpectedly, expression of *inhibin α* mRNA and DsRed decreased and eventually became undetectable during the culture process. Nevertheless, these results indicate that the TSC line at least partially retained the characteristics of Sertoli cells in a living organism.

### Optimizing the culture of type A spermatogonia

**Fig. 2:**
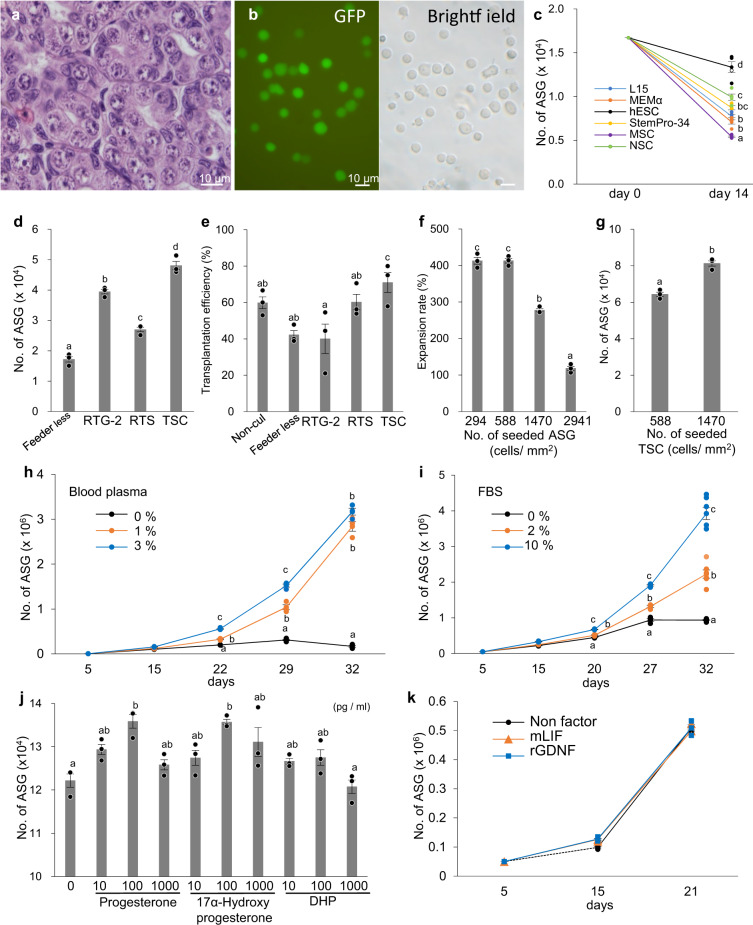
Optimization of culture conditions for trout ASGs. **a** Hematoxylin and eosin-stained histological section of an immature testis from a 12-month-old *vasa*-*gfp* transgenic rainbow trout used for experiments. **b** Fluorescent and bright field views of dissociated testicular cells from 12-month-old *vasa*-*gfp* transgenic fish. ASGs were specifically and strongly labeled with GFP. **c** The effect of six different types of culture medium (Supplementary Table 1) on the number of trout ASGs. **d** Effect of the feeder cell layer on the number of ASGs. Enriched ASGs were cultured on TSC, RTG-2, or rainbow trout spleen cell (RTS) lines with human embryonic stem cell medium. Feeder-free conditions were used as the control. **e** Effect of feeder cell layers on ASG transplantability. The percent of recipient fish with transplanted ASGs in their gonads at 20 dpt are shown as transplantation efficiency. **f** Effect of ASG seeding density on ASG growth in cultures. **g** Effect of TSC seeding density on number of ASGs. **h** Effect of trout blood plasma on number of ASGs in the presence of a TSC feeder layer in human embryonic stem cell medium supplemented with 10% FBS. **i** Effect of FBS on the number of ASGs in the presence of a TSC feeder layer in human embryonic stem cell medium supplemented with 1% trout blood plasma. **j** Effect of three progestins, **k** rat glial cell line-derived neurotrophic factor (rGDNF), and mouse leukemia inhibitory factor (LIF) in the presence of a TSC feeder layer in human embryonic stem cell medium supplemented with 10% FBS and 1% trout blood plasma on the number of ASGs. In **c**, **d**, **f**, **g**, and **j**, the number of ASGs was determined after 14 days of culture. All data are shown as the mean ± SEM (**c**, **k** *n* = 4; **d**, **e**, **f**, **g**, **h**, **j** *n* = 3; **i** *n* = 3 (5–27 days) and 6 (32 days)). Groups with different letters are significantly different based on one-way ANOVA followed by Tukey’s multiple comparisons test (*P* < 0.05) (**c**–**f** and **h**–**k**) and Student’s *t*-test (*P* = 0.0017) (**g**).

We also sought to identify an optimal culture medium for trout ASGs. GFP-labeled ASGs enriched from the immature testes (Fig. 2a, b) of *vasa*-*gfp* transgenic rainbow trout^15,16^ were cultured in six different types of medium (Supplementary Table 1) and changes in number of ASGs were monitored. Although increased ASGs were not observed under any tested culture medium, a human embryonic stem cell (hESC) medium improved ASG survival during the 2-week test period (Fig. 2c). The next step was to observe whether the established TSC line could serve as feeder cells to improve the ASG culture in human embryonic stem cell medium. Two other cell lines derived from rainbow trout [rainbow trout gonad-2 (RTG-2) and rainbow trout spleen (RTS)] were also tested. The TSCs most effectively supported an increment of ASG numbers among the three tested cell lines. Further, the number of ASGs increased by a maximum of 2.6-fold during the 2-week test period (Fig. 2d). ASG transplantation assays further showed that ASGs cultured on a TSC feeder layer possessed the highest transplantability among the three cell lines. This indicates that they had the greatest ability to migrate toward and be incorporated into recipient genital ridges after intraperitoneal transplantation (Fig. 2e). Further optimization of the seeding density of ASGs and TSCs revealed that 588 cells/mm^2^ and 1470 cells/mm^2^ were suitable for the initial seeding density (Fig. 2f, g).

To further enhance ASG proliferation in culture, we next examined the effects of soluble factors. Addition of trout blood plasma (1 or 3%; Fig. 2h) or FBS (2 or 10%; Fig. 2i) induced ASG proliferation in vitro in the presence of the TSC feeder layer in human embryonic stem cell medium. Simultaneous addition of trout blood plasma (1 or 3%) and 10% FBS effectively induced ASG proliferation in vitro (Fig. 2h, i). Addition of fish serum (0.25–3%), which is one of the soluble factors that induced ASG mitotic activity in our previous study^7^, was less effective than the trout blood plasma supplement. In addition, testicular somatic cells (fibroblast-like cells), which were carried over into the culture with the enriched ASG population, did not overgrow under the presence of the TSC feeder layer. Further attempts to identify the effective factors revealed that addition of progesterone (100 pg/ml) and 17α-hydroxyprogesterone (100 pg/ml), but not 17,20β-dihydroxy-4-pregnen-3-one (DHP), slightly, but significantly, enhanced ASG numbers maximally to ~1.2-fold greater than that in the control group (Fig. 2j). Addition of 11-ketotestosterone (1000 pg/ml), which is a major androgen in fish^13^, and estradiol-17β (100 pg/ml) that induced ASG mitotic activity in eel^13^ had no effects on ASG numbers (Supplementary Fig. 1). The addition of rat glial cell line-derived neurotrophic factor and mouse leukemia inhibitory factor had no effect during the 3-week test period (Fig. 2k). Based on the results from our present and previous studies, we developed the TS medium-3 culture medium (Supplementary Table 1) for the in vitro culture of ASGs.

### Characterization of in vitro-expanded ASGs

**Fig. 3:**
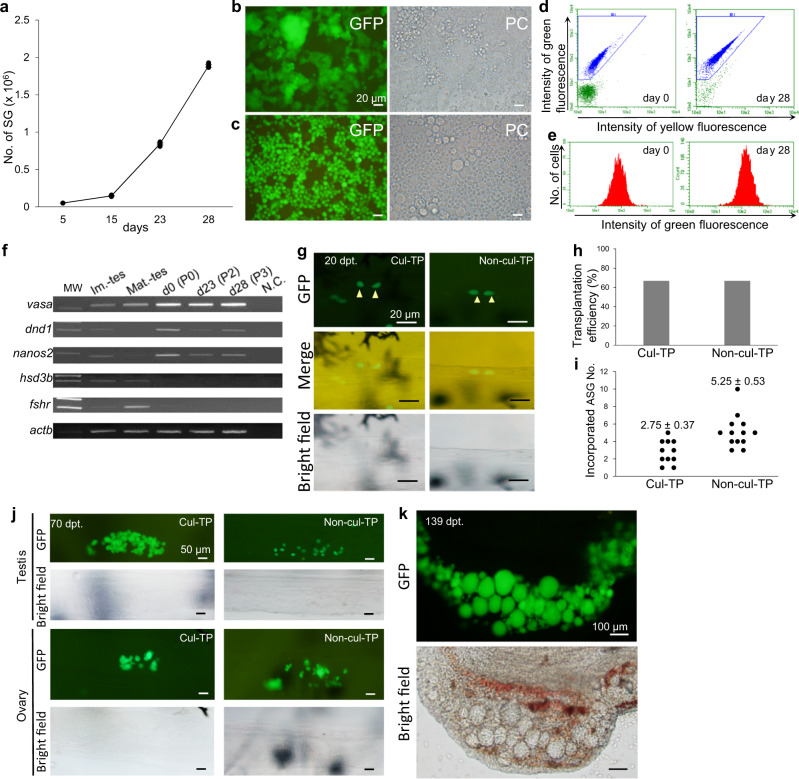
Characterization of in vitro-expanded ASGs. **a** Change in the number of ASGs in TS medium-3 in the presence of a TSC feeder layer. All data are shown as the mean ± SEM (*n* = 3). **b** Fluorescence (GFP) and phase-contrast (PC) micrographs of the 28-day-cultured ASGs. **c** Cultured ASGs after trypsin dispersion. **d** Fluorescence intensity of ASGs before (day 0) and after a 28-day culture period assessed by flow cytometry. The *x* axis indicates yellow fluorescing protein intensity (logarithmic scale) and the *y* axis indicates the GFP intensity (logarithmic scale). The gated region with a blue line indicates the ASG population. **e** Fluorescence intensity of ASGs. The *x* axis indicates the GFP intensity (logarithmic scale) and the *y* axis indicates cell counts (linear scale). **f** RT-PCR analysis of the expression of germ cell markers (*vasa*, *dnd1*, and *nanos2*) in ASGs. ASGs were isolated and analyzed from cultures at day 23 (d23) after 2 passages (P2) and day 28 (d28) after 3 passages (P3) by a flow cytometer. cDNA from immature testis (Im.-tes), mature testis (Mat.-tes), and non-cultured ASGs sorted by a flow cytometer (d0) were synthesized and used as controls in the analysis. Expression of *hsd3β* (Leydig cell marker) and *fshr* (Sertoli and Leydig cell marker) was also investigated. *actb* was used as an internal control. Reactions lacking the template (N.C.) were included as a negative control. **g** Micrographs of recipient genital ridges that received in vitro-expanded ASGs (Cul-TP) or freshly dispersed testicular cells (Non-cul-TP) at 20 dpt. Arrowheads indicate the ASGs that were incorporated into the recipient gonads. **h** The percentage of recipient fish carrying donor-derived ASGs in their genital ridges (TP efficiency) at 20 dpt. **i** The number of ASGs incorporated into recipient gonads at 20 dpt. **j** Micrographs of recipient gonads that received in vitro-expanded ASGs (Cul-TP) or freshly prepared testicular cells (Nn-cul-TP) at 70 dpt. The in vitro-expanded ASGs colonized in male (Testis) and female (Ovary) recipient gonads. **k** Micrographs of a female recipient gonad that received expanded ASGs. The oocytes derived from in vitro-expanded ASGs were observed at 139 dpt.

The combined use of the TSC feeder layer and TS medium-3 allowed us to stably expand the number of ASGs by 37.8-fold over the 28-day culture period (Fig. 3a). The expanded ASGs attached to or formed small clumps on the TSC feeder layer (Fig. 3b). Observation of trypsin-dispersed ASGs showed that the in vitro*-*expanded ASGs retained strong GFP expression, which mirrored the endogenous *vasa* gene expression level (Fig. 3c). Flow cytometry analysis showed that, even after several passages during the 28-day culture period, GFP intensity of the in vitro*-*expanded ASGs was similar to that of ASGs on the initial culture day (Fig. 3d, e). RT-PCR analysis demonstrated that during the 28-day culture period, in vitro*-*expanded ASGs expressed a subset of germ cell markers (*vasa*, *dnd1*, and *nanos2*; Fig. 3f). The expression of *fshr*, which is a Sertoli and Leydig cell marker, or *hsd3β*, which is a Leydig cell marker, was not detected in expanded ASGs (Fig. 3f). Furthermore, an intraperitoneal transplantation assay demonstrated that ASGs that were expanded in vitro for 28 days (Cul-TP group in Fig. 3g) had the ability to migrate toward and be incorporated into recipient genital ridges, similar to freshly prepared ASGs (Non-cul-TP group in Fig. 3g). No significant differences were observed between the Cul-TP and the Non-cul-TP groups in the percent of recipient fish having transplanted ASGs in their gonads (transplantation efficiency; Fig. 3h) or the number of ASGs incorporated into recipient genital ridges (Fig. 3i). Observation of the recipients at 70 days post-transplantation (dpt) revealed that the ASGs expanded in culture (Cul-TP group in Fig. 3j) could proliferate in and colonize the recipient gonads in both sexes similarly to freshly prepared ASGs (Non-cul-TP group in Fig. 3j). Furthermore, the in vitro*-*expanded ASGs were able to differentiate into oocytes in the female recipient gonads (Fig. 3k).

### Production of gametes from in vitro-expanded ASGs

**Fig. 4:**
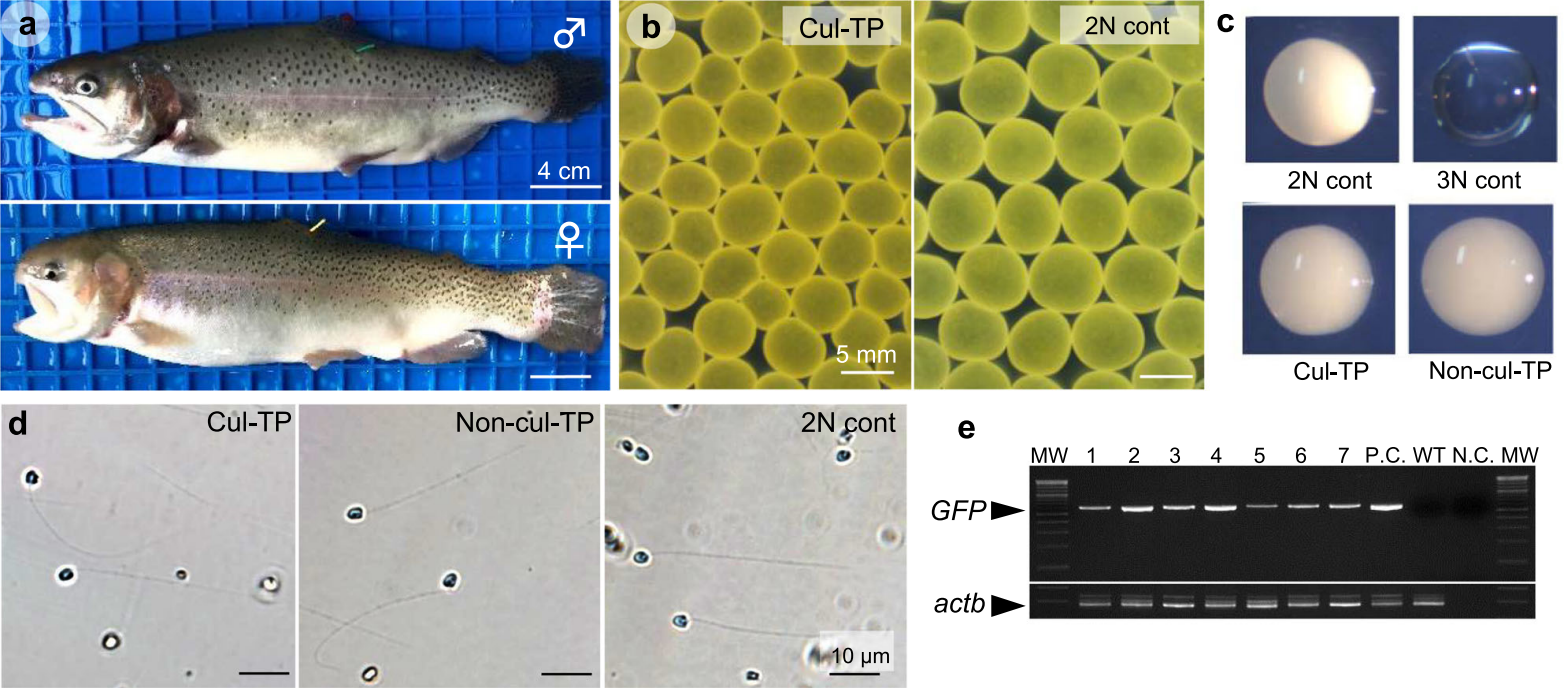
Production of functional eggs and sperm from in vitro-expanded ASGs by ASG transplantation. **a** Representative pictures of triploid male (♂) and female (♀) recipients that received in vitro-expanded ASGs. **b** Eggs produced by triploid female recipients that received expanded ASGs in culture (Cul-TP) or wild-type diploid female trout (2N cont). **c** Milt produced by wild-type diploid trout (2N cont), triploid trout (3N cont), male recipient trout that received in vitro-expanded ASGs (Cul-TP), or male recipient trout that received freshly prepared testicular cells (Non-cul-TP). **d** Micrographs of sperm produced by male recipient trout that received in vitro-expanded ASGs (Cul-TP), male recipient trout that received freshly prepared testicular cells (Non-cul-TP), or wild-type diploid trout (2N cont). **e** PCR analysis of milt produced by triploid male recipients (7 individuals, #1–7) with *gfp*-specific primers. *actb* was used as an internal control. Reactions using the DNA from *vasa-gfp* transgenic (P.C.) and wild-type (WT) trout were included as controls. Reactions lacking the template (N.C.) were also included as the negative control.

To examine whether in vitro*-*expanded ASGs maintained their ability to differentiate into functional gametes, 28-day expanded ASGs were transplanted into sterile triploid recipients^17^ that were reared until the maturation period. One year after transplantation, 66 of the 85 male recipients (77.6%) produced white-colored milt at a similar frequency to that of the 25 control recipients that received non-cultured ASGs and of which 22 (88%) produced milt. Due to space availability, 18 randomly selected males that produced white-colored milt were reared one additional year to undergo progeny tests with female recipients. At 2 years after the transplantation, 14 of 38 (36.8%) female and 7 of 7 (100%) male triploid recipients exhibited normal secondary sexual characteristics (Fig. 4a) and produced eggs and milt, respectively. Eleven males died before reaching 2 years of age and 11 out of 15 control females (73.3%), receiving non-cultured ASGs also produced eggs. Although the size of the eggs produced by triploid recipients that received in vitro*-*expanded ASGs was slightly smaller than those produced by normal diploid female trout (2N cont), the triploid recipient egg sizes were still within the normal range for rainbow trout^18^ (Fig. 4b). The milt produced by male triploid recipients that received in vitro*-*expanded ASGs was white in color and was almost indistinguishable from that of wild-type trout (2N cont) and male triploid recipients that received non-cultured ASGs (Non-cul-TP in Fig. 4c). Although the milt volumes (3.19 ± 0.71 ml) and total sperm number (1.2 ± 0.6 × 10^10^) produced by male triploid recipients was less than that of a triploid recipient that received non-cultured ASGs (5.3 × 10^10^ sperm in 7.38 ml milt), the values were still within the range for wild-type 2 N cont rainbow trout^18^ (Table 1). Optical microscopy further showed similar sperm morphology among male recipients and diploid controls (Fig. 4d). PCR analysis demonstrated the presence of the *gfp* gene in all milt produced by two-year-old triploid male recipients that received in vitro*-*expanded ASGs (Fig. 4e). No egg production was observed in any triploid fish that had not received transplants. Although some control triploid males produced extremely small amounts of milt, it did not possess developmental ability (Fig. 4c, 3N cont).

**Table 1:** Milt volume, sperm number, and egg number obtained from recipients receiving in vitro-expanded (Cul-TP) and non-cultured (Non-cul-TP) type A spermatogonia.

Donor cells	Sperm volume (μl)	No. of sperm (×10^10^)	No. of egg
Cul-TP ♂ (*n* = 7)	3190 ± 713.2	1.2 ± 0.6	—
Non-cul-TP ♂ (*n* = 1)	7380	5.3	—
Cul-TP ♀ (*n* = 8)	—	—	2569 ± 127
Non-cul-TP ♀ (*n* = 5)	—	—	2257 ± 174

**Fig. 5:**
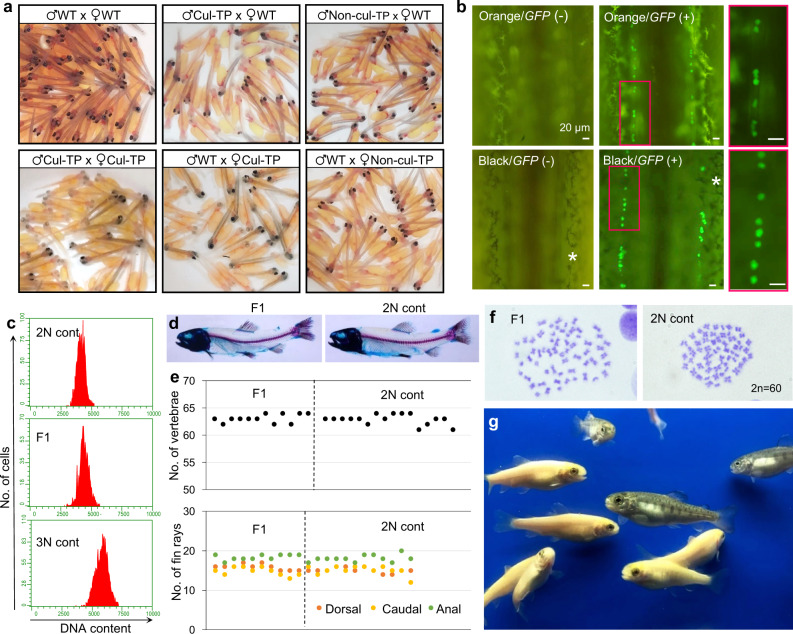
Production of in vitro-expanded ASGs-derived offspring using triploid recipients. **a** F1 offspring hatchlings produced by a cross-breeding experiment with wild-type (WT) diploid trout, triploid recipients that received in vitro-expanded ASGs (Cul-TP), and triploid recipients that received freshly prepared testicular cells (Non-cul-TP). **b** Fluorescence micrographs of the genital ridges of F1 offspring produced from triploid male or female recipients that received in vitro-expanded ASGs. As these ASGs were derived from dominant orange-colored (heterozygous, Orange/WT) *vasa*-*gfp* transgenic trout (heterozygous, GFP/−), F1 offspring exhibited four phenotypic patterns: orange body color with GFP-negative germ cells (Orange/Gfp−), orange body color with GFP-positive germ cells (Orange/Gfp+), black-pigmented body color with GFP-negative germ cells (Black/Gfp−), or black-pigmented body color with GFP-positive germ cells (Black/Gfp+). Higher magnification views of the red insets are also shown with red frames. **c** DNA contents of a wild-type diploid trout (2N cont), F1 juvenile (F1), and triploid trout (3N cont). **d** Representative picture of transparent skeleton specimens of an F1 offspring from triploid male/female recipients (F1) and a wild-type diploid trout (2N cont). **e** The number of vertebrae and dorsal, caudal, and anal fin rays in F1 offspring from triploid male/female recipients (F1) and wild-type diploid trout (2N cont). **f** Karyotype of an F1 offspring (F1; 2*n* = 60) and wild-type diploid trout (2N cont; 2*n* = 60). The F1 offspring possessed the same karyotype as that of donor trout (2*n* = 60). **g** Seven-month-old F1 offspring developed from expanded ASG-derived sperm and eggs.

Progeny tests were performed to examine the functionality of gametes derived from in vitro-expanded ASGs. Gametes from wild-type diploid trout and triploid recipients that received non-cultured ASGs were used as controls. The percent of fertilization, eyed eggs, hatching, and swimming-up rates did not show any significant differences between cultured ASG-derived and control offspring (Table 2). Transmission of the donor-derived haplotype to the F1 generation was confirmed by body color and green fluorescing germline cells (Fig. 5a, b; Table 2). In the F1 juveniles resulting from reciprocal fertilization between expanded ASG-derived sperm/eggs and wild-type diploid trout sperm/eggs, the occurrence frequencies of orange-colored fish and *vasa-gfp* (+) fish were around 50%. By contrast, when F1 juveniles were produced from cross-fertilization between expanded ASG-derived sperm and eggs, the occurrence frequencies were nearly 75% (Fig. 5a; Table 2). These results demonstrate that the donor-derived haplotypes of orange body color and *vasa*-*gfp* transgene were transmitted to offspring following Mendelian inheritance. DNA content analyses using flow cytometry revealed that all F1 juveniles produced from the cross-fertilization between expanded ASG-derived sperm and eggs were diploid (*n* = 15) and that none of the cells showed signs of aneuploidy (Fig. 5c). The number of vertebrae (*n* = 12) and fin rays (dorsal, caudal, and anal, *n* = 10) were similar between cultured ASG-derived and wild-type diploid offspring (Fig. 5d, e). Karyotype analysis of F1 juveniles (*n* = 3) created by expanded ASG-derived sperm and egg cross-fertilization indicated a normal diploid karyotype consisting of 60 chromosomes (Fig. 5f). The F1 juveniles developed from in vitro-expanded ASG-derived sperm and eggs were morphologically normal and grew up normally (Fig. 5g).

**Table 2:** Early survival and their phenotype of offspring obtained by mating of recipients.

Eggs	Sperm	Combination	Egg number	Fertilized (%)	Eyed (%)	Hatching (%)	Swimming up (%)	Orange-colored (%)	GFP (%)
Cul-TP^a^	Wild type	*N* = 7	468.0 ± 24.3	95.2 ± 2.1	86.7 ± 2.4	68.7 ± 3.3	68.5 ± 3.4	50.6 ± 1.2	47.1 ± 1.8
Wild type	Cul-TP^a^	*N* = 5	190.4 ± 11.5	97.5 ± 1.0	89.4 ± 2.6	85.7 ± 3.4	83.6 ± 4.1	47.4 ± 2.4	53.0 ± 2.0
Cul-TP^a^	Cul-TP^a^	*N* = 5	444.6 ± 15.1	96.7 ± 1.6	84.7 ± 8.0	68.5 ± 7.8	67.6 ± 7.6	77.7 ± 2.4	78.8 ± 2.8
Non-Cul-TP^a^	Wild type	*N* = 5	387.6 ± 21.2	97.5 ± 1.7	86.1 ± 4.4	76.6 ± 6.7	75.6 ± 6.5	49.2 ± 0.9	51.5 ± 1.0
Wild type	Non-Cul-TP^a^	*N* = 1	318.0 ± 0.0	87.5 ± 0.0	84.6 ± 0.0	80.5 ± 0.0	79.2 ± 0.0	51.6 ± 0.0	45.8 ± 0.0
Non-Cul-TP^a^	Non-Cul-TP^a^	*N* = 4	507.5 ± 43.4	95.8 ± 0.0	83.6 ± 4.7	68.4 ± 8.0	67.3 ± 7.8	72.8 ± 1.0	70.0 ± 2.9
Wild type	Wild type	*N* = 3	208.0 ± 4.2	95.8 ± 2.4	92.0 ± 4.1	90.1 ± 3.8	89.9 ± 3.9	0.0 ± 0.0	0.0 ± 0.0

^a^Cul-TP and Non-cul-TP represents gametes obtained from recipients that were transplanted with in vitro-expanded and non-cultured type A spermatogonia, respectively.

## Discussion

This study successfully established a novel method to effectively expand ASGs of rainbow trout by using Sertoli cells as a feeder layer. Notably, the transplantation assay demonstrated that the expanded ASGs showed a high level of stemness. Furthermore, the ASGs possessed sexual plasticity and could differentiate into functional eggs and sperm. To the best of our knowledge, this is the first study to produce functional eggs derived from in vitro-expanded cells in any teleost species.

Cryopreservation of fish eggs or embryos is technically still not feasible in fish^3^, but a combination of GSC cryopreservation and subsequent transplantation is a powerful alternative^4,5,18^ for long-term preservation of genetic resources of endangered fish species. However, we often face a difficulty that only juvenile fish possessing extremely small gonads can be harvested during field sampling, making it difficult to supply enough GSCs for cryopreservation and transplantation. Further, some endangered fish species that lack adequate nourishment due to the shrinkage or destruction of their habitats also carry extremely small gonads. In the method developed in this study, ASGs could be amplified by 40 times within a month. Therefore, even though we consider the reduced transplantation efficiency (male recipients that received in vitro-expanded ASGs (77.6%) versus freshly prepared ASGs (88%) and female recipients that received in vitro-expanded ASGs (36.8%) versus freshly prepared ASGs (73.3%)), the quantities of recipients producing donor-derived gametes per unit donor ASGs increase 35-fold and 20-fold more in male and female recipients, respectively. Therefore, the method of in vitro expansion of ASGs established in this study is a breakthrough for these situations. Combination of in vitro expansion, cryopreservation^18^, and transplantation^11,19^ of ASGs can be utilized to rescue fish species on the verge of extinction.

During spermatogenesis, germ cells are supported by appropriate microenvironments created primarily by Sertoli cells. In fish, spermatogenesis progresses in testicular cysts and germ cells are encapsulated by Sertoli cells starting at the single ASG stage^13^. Sertoli cells then change their physiological characteristics to support the corresponding stages of germ cell differentiation. Therefore, Sertoli cells that specifically nourish ASGs can be easily obtained in fish. Furthermore, under-yearling salmonid juveniles, including rainbow trout, only possess ASGs in their testes and the exclusive role of the Sertoli cells is to nourish ASGs. These characteristics make Sertoli cells a suitable candidate to be used as a feeder layer to support ASGs in vitro. This is one potential reason why our Sertoli feeder layer effectively maintained mitotic trout ASGs (most likely spermatogonial stem cells carrying transplantability) in vitro. Another advantage of our system was that fish Sertoli cells are mitotically active even in adults^14^, making it possible to establish a cell line without any immortalization treatments by introducing foreign genes, such as SV-40 large T-antigen^20^ or telomere reverse transcriptase^21^. As these unique characteristics of Sertoli cells are well conserved in various teleosts, the strategy established in this study may set the gold standard for performing in vitro expansion of fish ASGs that exhibit stemness and sexual plasticity.

Although there are several reports describing germ cell culture in various fish species, including medaka^6^, zebrafish^8,9^, and tilapia^10^ none have simultaneously reported evidences that the germ cells can exponentially expand in vitro and the ability of the expanded germ cells to differentiate into functional gametes after transplantation into recipients. Previously, we also developed a culture condition that allowed the maintenance of trout ASGs for 1–2 months^7,22^. However, in vitro ASG expansion was not observed under these conditions and the ASGs eventually disappeared from the cultures during consecutive culture passage^7,22^. To overcome this defect, the current study focused on reproducing the fish testis microenvironment in vitro using a TSC feeder layer derived from rainbow trout Sertoli cells and a medium containing rainbow trout blood plasma. Although these culture conditions did not completely reconstruct the testis microenvironment, their effects were quite striking. In addition, use of human embryonic stem cell medium steadily improved the ASG cultures and FBS was shown to be essential component for ASG expansion in vitro. Thus far, it is still unclear which components in trout blood plasma and FBS induce ASG proliferation in vitro. As ASG expansion was not observed without the TSC feeder layer, even in the presence of a similarly supplemented TS medium-3, it is possible that some trout blood plasma and FBS components promoted production of growth factors in the TSC line, which in turn induced ASG expansion in a paracrine manner. The ultimate goal of this series of studies is the establishment of a stable and transplantable germ cell line. This may be accomplished by more precisely reproducing the trout testis microenvironment using improved TSC with more similar molecular and physiological characteristics to Sertoli cells in a living organism.

Overgrowth of testicular somatic cells (fibroblast-like cells) that were carried over into cultures with the enriched ASG population was a major issue in our previous studies^7,22^. As this overgrowth occurred in culture medium containing relatively high FBS concentrations (>10%), we used a medium containing 1% FBS to suppress somatic cell overgrowth. In the present study, however, somatic cell overgrowth was not observed in the presence of the TSC feeder layer, even in medium containing >10% FBS. This finding suggests that proliferation of fibroblast-like cells in cultures can be inhibited by factors produced by TSCs or the paucity of an available solid surface area in the culture plate for growth.

In vitro ASG expansion may simplify the production of fish seedlings with high commercial value. Whenever seedlings of large fish species, such as bluefin tuna or giant grouper, are produced using germ cell transplantation^5^, securing a supply of donor ASGs is one of the most difficult and costly steps. However, using the in vitro ASG expansion protocol established in this study would allow for production of seedlings using donor ASGs prepared in petri-dishes and small-bodied recipient fish with a short generation time in place of giant donor fish. This method would allow for simplified fish seedling production of the donor-species that can be performed in small facilities at lower costs. In addition, the in vitro ASG culture system established in this study provides a platform for studying spermatogenesis, especially the interaction of GSCs or ASGs with Sertoli cells. This is possible not only in teleosts, but also in all anamniotes, in which spermatogenesis progresses in testicular cysts surrounded by Sertoli cells.

## Methods

### Fish

A transgenic rainbow trout strain carrying the *DsRed* gene driven by *inhibin* α gene-regulatory elements (*inhibin*-*DsRed* transgenic trout) was created and used for establishment of the TSC line. Briefly, template genomic DNA was purified from trout adipose fin and the *inhibin* α gene promotor region was amplified by PCR using a specific primer set (see Supplementary Table 2). The resulting fragment containing the 2.2 kb 5′-upstream region was cloned into the *Hin*dIII and *Bam*HI sites of pDsRed2-1 (Takara Bio, Inc., Shiga, Japan). A total of 2 nl of the plasmid DNA (50 ng/μl) was delivered into the cytoplasm of 1-cell-stage embryo between 3 and 7 h after fertilization using a microinjector (IM-9B, Narishige)^23^. Another transgenic rainbow trout strain carrying the *gfp* gene driven by *vasa* gene-regulatory elements (*vasa*-*gfp* transgenic trout)^15,16^ was used for all ASG culture and transplantation experiments. Because the early stage of germline cells (e.g., primordial germ cells, ASGs, oogonia, and oocytes), but not somatic cells, were labeled with GFP in the transgenic strain, changes in the number, morphology, and behavior of these germ cells could be clearly monitored in vitro and in vivo. Triploid rainbow trout hatchlings were produced by heat shock treatment (28 °C for 15 min) started at 15 min after fertilization^19^ and used as recipients for the ASG transplantation assay. Nontransgenic (Oizumi strain) and transgenic fish used in this study were raised and maintained under 10.5 °C spring water in the Oizumi research station of Tokyo University of Marine Science and Technology (TUMSAT) in Yamanashi prefecture, Japan. All described procedures were carried out in accordance with the Guide for the Care and Use of Laboratory Animals from TUMSAT and approved by IACUC of TUMSAT.

### Histological analysis and in situ hybridization

Isolated immature testes were fixed in Bouin’s fixative, dehydrated, and embedded in paraplast embedding media (Leica Biosystems, Wetzlar, Germany). The specimens were then cut into 5 μm sections using a microtome (Leica Biosystems) and the hydrated sections were stained with hematoxylin and eosin solution. For in situ hybridization, digoxigenin-labeled sense and antisense probes were synthesized from a partial fragment of *inhibin* α cDNA (nucleotides 97–1059 of *inhibin*). The sections were incubated with a hybridization solution of 50 μg/ml tRNA, 50% formamide, 5 × SSC (pH 4.5), 50 μg/ml heparin, 1% SDS and 1 μg/ml probe at 65 °C for 18 h^24^. The sections were rinsed twice in 5 × SSC/50% formamide at 65 °C for 30 min, three times in 2 × SSC/50% formamide at 65 °C for 30 min, 1 × SSC/25% formamide/1 × TBST at 65 °C for 10 min, three times in 1 × TBST at room temperature for 5 min, lastly in blocking solution (Roche) at room temperature for 1 h. The sections were then incubated with the Fab fragment of an anti-DIG–alkaline phosphatase-conjugated antibody (Roche), diluted to 1:2000 with blocking solution, for 16 h at 4 °C. Color reaction was performed with the nitroblue tetrazolium (NBT; Roche) and 5-bromo-4-chloro-3-indolyl phosphate (BCIP; Roche).

### Microscopy

Immature testes isolated from 13-month-old *inhibin*-*DsRed* transgenic trout and nontransgenic trout were observed and photographed under a fluorescence stereomicroscope (Olympus, MVX-10 equipped with a U-MWIG2 filter and DP-73). Immature testes of *vasa*-*gfp*/*inhibin*-*DsRed* double transgenic trout were observed using confocal microscopy (Olympus, FV1000-D equipped with U-MWIG2 filter) to examine localization of DsRed in the testes. Cell nuclei were stained with Hoechst 33342 (Dojindo, Kumamoto, Japan). Dispersed testicular cells, isolated and cultured Sertoli cells, and ASGs were observed under an inverted fluorescence microscope (Olympus, CK-40 and IX-71 equipped with U-NWUB2).

### Isolation and culture of Sertoli cells

Immature testes of 15-month-old *inhibin*-*DsRed* transgenic trout were dissociated with a trypsin solution (W-trypsin solution) containing 0.43 U/ml trypsin (Worthington Biochemical Corporation, Lakewood, NJ, USA), 1 mM CaCl_2_, 5% FBS (ThermoFisher Scientific, Massachusetts, USA), and 40 U/ml DNase I (Sigma-Aldrich) in 1× PBS (pH 8.2) at 10 °C for 2.5 h. In a previous study, the FBS in this reaction was confirmed not to inhibit enzymatic activity under this condition and to improve testicular cell survival. Non-dissociated cell clumps were eliminated by filtration through a 42-μm-pore size nylon screen (NBC Industries, Tokyo, Japan). Subsequently, Sertoli cells (DsRed+) were isolated using a flow cytometer (MoFlo XDP Cell Sorter equipped with Summit v5.4, Beckman Coulter, California, USA) and seeded at a concentration of 53 cells/mm^2^ in 0.1% gelatin-coated 96-well plates. The effect of fish serum (0, 0.25, 0.5, 1, 2, or 4%; salmonid serum “SeaGrow” East Coast Bio, Maine, USA) on Sertoli cell growth was investigated using ERDF medium (pH 7.8; Kyokuto Pharmaceutical Industrial Co., LTD, Japan) supplemented with 10 mM HEPES (Sigma-Aldrich) and antibiotics (50 μg/ml ampicillin, 50 U/ml penicillin, and 50 μg/ml streptomycin; FUJIFILM Wako Pure Chemical Corporation). The effect of FBS (0, 1, 2, 4, 6, 8, 10, or 15%) was tested in the presence of 0.25% fish serum in the same medium. Sertoli cells were cultured for 14 days and their numbers were determined using flow cytometry (Guava easyCyte equipped with Cytosoft 5.3; Merck Millipore, Massachusetts, USA). To evaluate effectiveness of the optimized culture conditions, isolated Sertoli cells were cultured under the optimized culture medium (ERDF medium supplemented with 10 mM HEPES, 6% FBS, and 0.25% fish serum) or a standard culture medium for salmonids (Hank’s MEM supplemented with 25 mM HEPES and 5% FBS) for 7 weeks. Cell numbers were determined once a week using flow cytometry.

### Feeder cells

RTG-2 was purchased from ATCC (Summit Pharmaceuticals International, Tokyo, Japan) and maintained according to the instructions. The rainbow trout spleen cell line was established in our laboratory. Spleens isolated from 10-month-old rainbow trout were cut into small fragments (5 mm ×5 mm) and directly plated on plastic culture flasks (25 ml). The cells resembled to epithelial cells in cultures were maintained in L-15 medium (pH 7.8; ThermoFisher Scientific) containing 25 mM HEPES, 10% FBS, and antibiotics for more than 1 year with multiple passages (>50 times). The TSC line was cultured at 18 °C in the described optimized medium. Cells were passaged at 70–80% confluence. To use these cell lines as feeder cells for ASG culture, proliferation activity was inhibited by treatment with 10 µg/ml mitomycin C (Nakarai Tesque, Kyoto, Japan) and cells were seeded onto 0.1% gelatin-coated plates at a concentration of 1470 cells/mm^2^ in a 96-well plate.

### ASG enrichment and culture condition optimization

ASGs were enriched from the immature testes of *vasa*-*gfp* transgenic trout by a differential plating method^7,22^. To expand ASGs in cultures, the effects of different types of culture medium (Supplementary Table 1) on the number of ASGs was first tested. Second, the effects of feeder cells (see above), the ASG seeding concentration (294, 588, 1470, or 2941 cells/mm^2^) and the feeder cell seeding concentration (588 or 1470 cells/mm^2^) on number of ASGs were examined under the selected optimal culture medium. Third, the effects of trout blood plasma (0, 1, or 3%, prepared from immature male trout), FBS (0, 2, or 10%), three progestins (10–1000 pg/ml of progesterone, 17α-hydroxyprogesterone, or 17,20β-dihydroxy-4-pregnen-3-on; Sigma-Aldrich), 11-ketotestosterone (1000 pg/ml; Chemieliva), estradiol-17β (100 pg/ml; Sigma-Aldrich), and growth factors (100 ng/ml of rat glial cell line-derived neurotrophic factor, R&D Systems, Minnesota, USA; 1000 U/ml of recombinant mouse leukemia inhibitory factor, Merck Millipore) were tested in the presence of the optimal feeder cells using the selected optimal culture medium. For long-term culture of ASGs, enriched ASGs were cultured on TSC feeder layers at 18 °C in a 5% CO_2_ atmosphere in an optimized medium (TS medium-3; Supplementary table 1). The number of ASGs and GFP intensity were determined using flow cytometry (Guava easyCyte). Cultures were passaged every 5 or 6 days at 70–80% confluence with Accutase (ThermoFisher Scientific). To remove somatic cells and enrich ASGs, differential plating was performed for every passage. Briefly, the collected cells were seeded onto a new plate and maintained in TS medium-3 containing 40 µM Z-VAD-FMK (Peptide Institute, Inc., Osaka, Japan). Sixteen h after seeding, the culture medium was replaced with plain TS medium-3 without Z-VAD-FMK. Because most of the gonadal somatic cells transferred from the original culture plate were tightly attached to the bottom of the new culture plates, whereas ASGs were either suspended or weakly adhered to the bottom of the wells, the ASGs could be harvested by gentle pipetting and seeded onto a new plate with a TSC feeder layer.

### RT-PCR

The TSC line was harvested and RNA was extracted using a RNeasy mini kit (Qiagen, Hilden, Germany). cDNA was synthesized using a PrimeScript™ 1st-strand cDNA Synthesis Kit (Takara Bio Inc.). Expression of a subset of typical Sertoli cell markers (*sox9b*, *wt1*, *arb*, and *clu*), a marker for Sertoli and Leydig cells (*fshr*), and a Leydig cell marker (*hsd*3*b*) were examined by RT-PCR analysis. The cDNA of immature testes was similarly prepared and used as a positive control. For gene expression analysis of cultured ASGs, ASGs (GFP+) were isolated by a flow cytometer (Beckman Coulter) after being cultured for 0, 23, and 28 days and the cDNA was synthesized as described above. cDNA of immature testes and mature testes were similarly synthesized as controls. Expression of a subset of germ cell markers (*vasa*, *dnd1*, and *nanos2*), a Sertoli and Leydig cell marker (*fshr*), and a Leydig cell marker (*hsd*3*b*) were then examined by RT-PCR analysis. Expression of *β-actin* (*actb*) was used as an internal control for all RT-PCR analyses. All primer sequences used in this study are listed in Supplementary Table 2.

### Transplantation assay

ASGs derived from 12- to 15-month-old dominant orange-colored (heterozygous, orange/black; OR/wt) *vasa*-*gfp* transgenic trout (heterozygous, *vasa*-*gfp*/−) were cultured and used for transplantation assays. Approximately 15,000 ASGs from cultured or freshly dispersed testicular cells were microinjected into the peritoneal cavity of each nontransgenic triploid hatchling (30 days old) by using a microinjector (IM-9B, Narishige) under a stereomicroscope (SZX-10, Olympus)^11^. In each trial, testes from more than 10 individual fish were pooled and used to avoid individual variation in donor trout. Some of the recipients were dissected and their gonads were observed under a fluorescence microscope at 20, 70, and 139 dpt. The percentage of recipients possessing incorporated ASGs in their genital ridges (transplantation efficiency) and the total number of incorporated ASGs in each recipient were determined at 20 dpt.

### Progeny tests

Morphology of the obtained eggs and sperm were examined under a microscope (Olympus, MVX-10 and BX-51). The total sperm number was estimated by multiplying the spermatozoa concentration by the total milt volume. To confirm the production of sperm derived from transplanted ASGs (GFP+), total genomic DNA was extracted from the semen of 2-year-old recipients and PCR analysis was performed with a *gfp*-specific primer set (Supplementary Table 2). To evaluate the production of offspring derived from cultured ASGs (OR/wt and GFP/−), eggs and sperm produced by triploid recipients were used for fertilization with sperm and eggs obtained from wild-type diploid trout or triploid recipients. The phenotype of F1 offspring would be expected to be 50% orange body color (OR/wt) and 50% *vasa*-*gfp* (+) in cases where recipients were mated with wild-type counterparts or 75% orange body color (25% OR/OR and 50% OR/wt) and 75% *vasa*-*gfp* (+) in cases where male recipients were mated with female recipients following Mendelian inheritance. Gametes from wild-type diploid trout and triploid recipients that received non-cultured ASGs were used as controls. The percent of fertilization, eyed eggs, and hatching were recorded. The germline transmission rate was calculated from the body color of the offspring (orange or black) and the presence or absence of GFP-positive germ cells.

### Transparent skeleton specimen

Fish were fixed with 10% formalin for 7–10 days and their cartilages were stained for 30 min with 1 mg/ml alcian blue 8GX solution (Sigma-Aldrich) diluted in 70% ethanol and 30% glacial acetic acid. The specimens were then hydrated in a degraded ethanol series (95, 75, 50, and 25%) and immersed in 30% sodium borate solution containing 0.1 g/ml trypsin (ThermoFisher Scientific) for 16–24 h at 37 °C. After washing with 0.5% aqueous KOH, the endoskeletons were stained for 12 h with 20% Alizarin Red S (FUJIFILM Wako Pure Chemical Corporation) solution containing 0.4% KOH. The specimens were then immersed in a series of glycerin-0.5% KOH solutions (25, 50, and 75% glycerin) for several days each and preserved in pure glycerin containing 0.025% sodium azide. The numbers of vertebrae and fin rays were determined under a stereomicroscope (SZX-10, Olympus).

### Karyotype analysis

Phytohemagglutinin-L solution (0.1% in 1X PBS; Sigma-Aldrich) was injected at a dose of 0.1 ml/20 g of body weight and 0.05% colchicine solution (ThermoFisher Scientific) was injected 16 h later at a dose of 0.1 ml/20 g of body weight. Six h after the colchicine injection, the kidneys were isolated and dissociated with W-trypsin solution and incubated in hypotonic 0.075 M KCl (ThermoFisher Scientific) solution for 1 h. The resulting cells were fixed with 25% acetic acid and 75% methanol. Cells were then dropped on warmed slides and dried completely and stained with Giemsa stain solution (FUJIFILM Wako Pure Chemical Corporation). The chromosomes were photographed and counted under an Olympus BX53 microscope equipped with DP-73 (Olympus).

### Ploidy analysis

Trout peripheral bloods were collected with a needle containing 0.1% heparin solution (FUJIFILM Wako Pure Chemical Corporation). After fixation with chilled 70% ethanol at 4 °C, blood samples were centrifuged at 1000 rpm at 4 °C for 10 min. The collected blood-cell pellets were stained with propidium iodide solution (100 µg/ml; Dojindo) containing 35 µg/ml RNase A (Sigma-Aldrich) for 16 h at 4 °C. The fluorescent intensity of the stained cells was determined using flow cytometry (Guava easyCyte).

### Statistics and reproducibility

All data are presented as the mean value ± standard error of the mean (SEM). Statistical significance was determined using Student’s *t*-test for comparisons between two groups. For comparisons among three or more groups, statistical significance was determined using a one-way ANOVA followed by Tukey’s multiple comparisons test using a statistical significance level of *P* < 0.05. All analyses were carried out by GraphPad Prism 7 (GraphPad Software, California, USA).

### Reporting summary

Further information on research design is available in the Nature Research Reporting Summary linked to this article.

## Supplementary information

Supplementary Information

Description of Additional Supplementary Files

Supplementary Data 1

Supplementary Data 2

Reporting Summary

## Data Availability

The data that support the findings in this study are available upon reasonable request from the corresponding author. The source data underlying the graphs and gel electrophoresis are provided in Supplementary Data 1 and 2.
