# Supplementary material for: Production of functional eggs and sperm from in vitro-expanded type A spermatogonia in rainbow trout
Source: Commun Biol. 2020 Jun 15;3:308. doi: 10.1038/s42003-020-1025-y (PMC7296041; doi:10.1038/s42003-020-1025-y)
Supplement: Supplementary file 1 — Supplementary Information [file 42003_2020_1025_MOESM1_ESM.pdf]

Supplementary table 1. Components of cell culture medium.

Medium name	L15	MEMα	hESC	StemPro-34	MSC	NSC	TS-3
**Basal medium**	L15	MEMα	DMEM/F12+GlutaMAX	StemPro-34 SFM	MSC SFM Basal Medium	KnockOUT DMEM/F12	DMEM/F12+GlutaMAX
**HEPES (25 mM)**	+ (pH 7.8)	-	-	-	-	-	-
**Fetal bovine serum (%)**	2	2	2	2	2	2	10
**Bovine serum albumin (%)**	0.5	0.5	1.8	0.5	0.5	0.5	1.8
**L-aspartic acid (20 μg/ml)**	+	+	+	+	+	+	-
**L-cystin (20 μg/ml)**	+	+	+	+	+	+	-
**L-prolin (20 μg/ml)**	+	+	+	+	+	+	-
**L-glutamic acid (20 μg/ml)**	+	+	+	+	+	+	-
**Ascorbic acid (50 μM)**	+	+	+	+	+	+	+
**Salmonids serum (0.25%)**	+	+	+	+	+	+	-
**Rainbow trout blood plasma (1%)**	-	-	-	-	-	-	+
**2-Mercaptoethanol (**μM**)**	50	50	55	50	50	50	55
**human bFGF (**ng/ml**)**	1	1	8	1	1	1	8
**Rainbow trout embryonic extract (1 μg/ml)**	+	+	+	+	+	+	-
**Chemically Defined Lipid Concentrate (0.1%)**	+	+	+	+	+	+	+
**Z-VAD-FMK (40 μM)**	-	-	-	-	-	-	+
**Progesterone (100pg/ ml)**	-	-	-	-	-	-	+
**Penicillin (50 U/ml)**	+	+	+	+	+	+	+
**Ampicillin (50 μg/ml)**	+	+	+	+	+	+	+
**Streptomycin (50 μg/ml)**	+	+	+	+	+	+	+
**Stempro®hESC SFM Growth Supplement**	-	-	+	-	-	-	+
**Stempro®MSC SFM Supplement**	-	-	-	-	+	-	-
**StemPro-34 Nutrient Supplement**	-	-	-	+	-	-	-
**StemPro Neural Supplement**	-	-	-	-	-	+	-

Supplementary table 2. Primer sequences used for PCRs.

Primer name	Primer sequence
**inhibinP-FW**	5’-TGAAAAGCTTAGTATTTGGTGAACAACA
**inhibinP-RV**	5’-TGAAGGATCCCCAGTCTGCATGGTTCAGAT
**vasa-FW**	5'-TCTTCAGAGAGATGGGGCAAGTCATC
**vasa-RV**	5'-TCCCATATCCAGGACCACACGCACATT
**dnd1-FW**	5'-AGATGTGGCTGCAGGAGACT
**dnd1-RV**	5'-GTTCATCATGAGGCGGAACT
**nanos2-FW**	5'-GAATTCGATCGGGACTGAGA
**nanos2-RV**	5'-CAGAATGGGGCAGATGACTT
**hsd3b-FW**	5'-TTGGACTGGGCCATGTCTCT
**hsd3b-RV**	5'-ATGCTGCTGGTGTAGATGAAGGA
**fshr-FW**	5'-TGAAGCCATTCTGGACACTT
**fshr-RV**	5'-TGTTAGCACAATGGCAGTGA
**sox9b-FW**	5'-AGAGGTCAGCCCTTAGCACA
**sox9b-RV**	5'-GAGCCGTTGTTCCCAAGATA
**arb-FW**	5'-TACTGTCACCGAAACCTTGGA
**arb-RV**	5'-TGGTTGTGCAGGCAGAATTGGA
**clu-FW**	5'-AAAGCCTTCCGGTATGACCT
**clu-RV**	5'-TCAAACCTCCTCAGCTGCTT
**actb-FW**	5'-ACTACCTGATGAAGATCCTG
**actb-RV**	5'-TTGCTGATCCACATCTGTTG

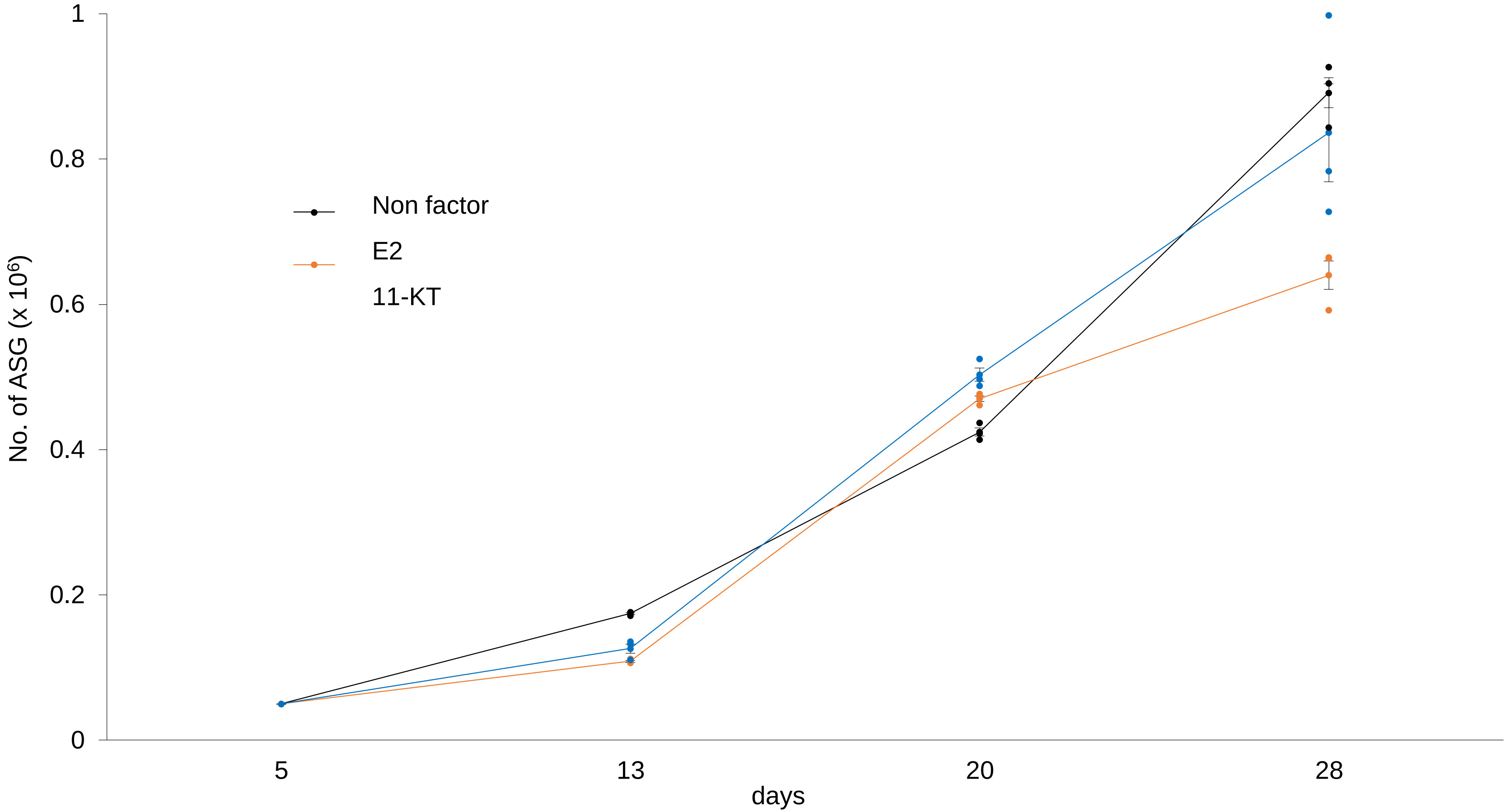

Supplementary figure 1. Effect of sex steroids on the number of ASGs.
Effect of 11-ketotestosterone (1,000 pg/ml) and estradiol-17β (100 pg/ml) in the presence of a TSC feeder layer in human embryonic stem cell medium supplemented with 10% FBS and 1% trout blood plasma on the number of ASGs. Data are shown as the mean ± SEM (n=3).
