# Supplementary material for: Production of functional eggs and sperm from in vitro-expanded type A spermatogonia in rainbow trout
Source: Commun Biol. 2020 Jun 15;3:308. doi: 10.1038/s42003-020-1025-y (PMC7296041; doi:10.1038/s42003-020-1025-y)
Supplement: Supplementary file 2 — Description of Additional Supplementary Files [file 42003_2020_1025_MOESM2_ESM.pdf]

Description of Additional Supplementary Files

File Name: **Supplementary Data 1**
Description: Source data for graphs

File Name: **Supplementary Data 2**
Description: Source data for gels
