# Supplementary material for: Production of functional eggs and sperm from in vitro-expanded type A spermatogonia in rainbow trout
Source: Commun Biol. 2020 Jun 15;3:308. doi: 10.1038/s42003-020-1025-y (PMC7296041; doi:10.1038/s42003-020-1025-y)
Supplement: Supplementary file 4 — Supplementary Data 2 [file 42003_2020_1025_MOESM4_ESM.pptx]

## Slide 1
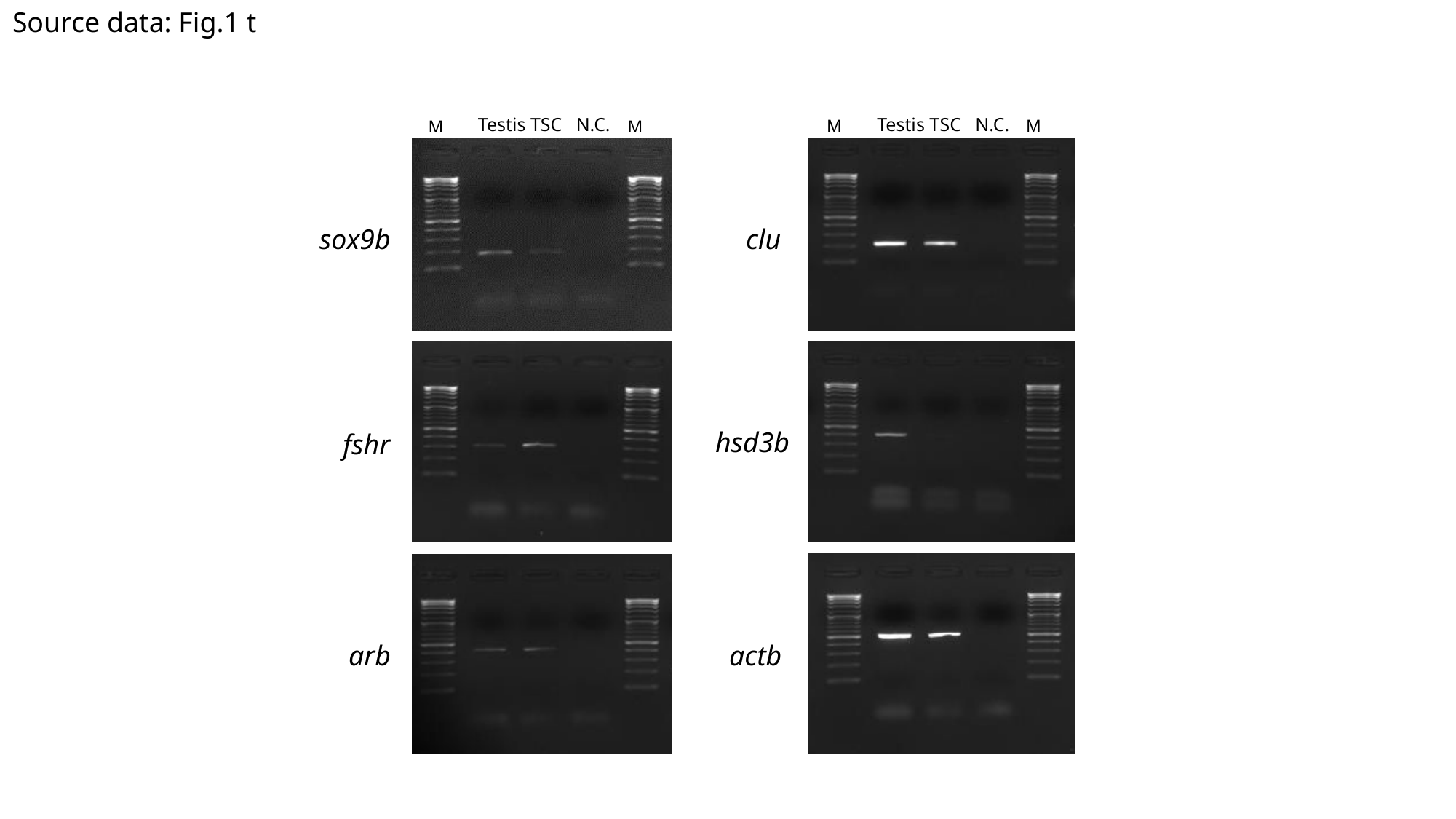

Source data: Fig.1 t
Testis TSC N.C.
Testis TSC N.C.
M
M
M
M
sox9b
clu
hsd3b
fshr
arb
actb

## Slide 2
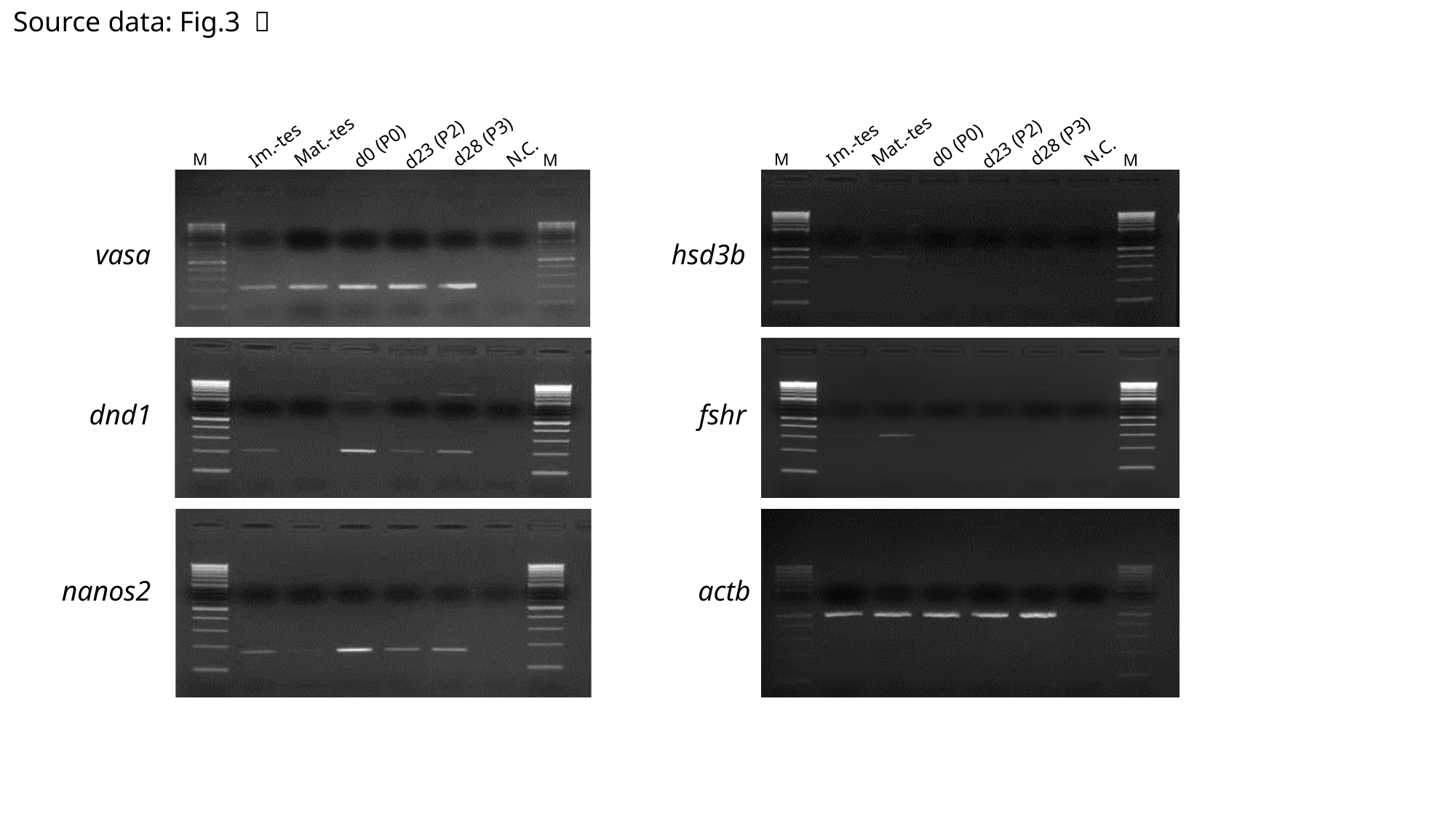

Source data: Fig.3 ｆ
d28 (P3)
Mat.-tes
d23 (P2)
Im.-tes
d0 (P0)
N.C.
d28 (P3)
Mat.-tes
d23 (P2)
Im.-tes
d0 (P0)
N.C.
M
M
M
M
vasa
hsd3b
dnd1
fshr
nanos2
actb

## Slide 3
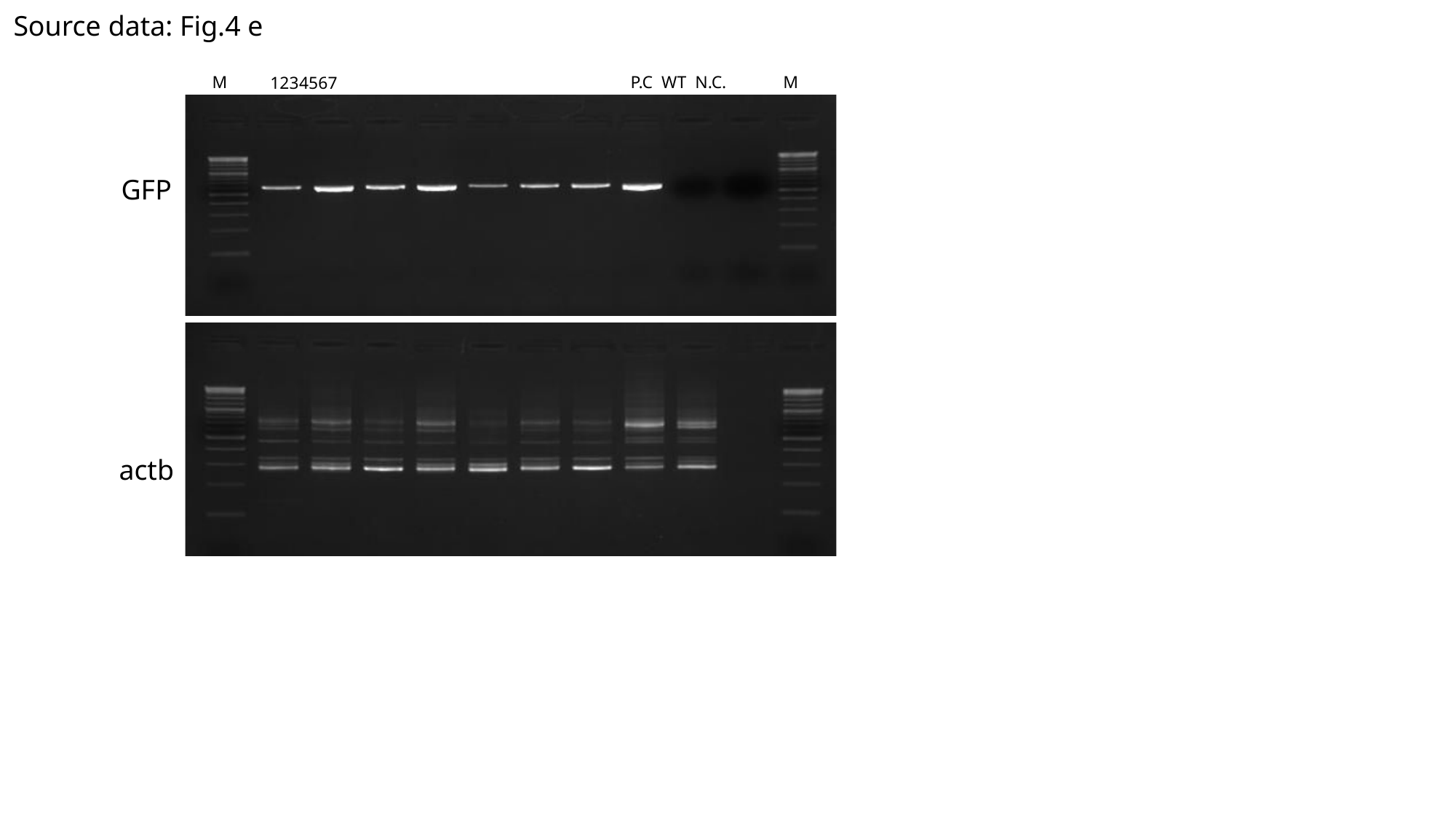

Source data: Fig.4 e
M
P.C WT N.C.
M
1234567
GFP
actb
