# Supplementary material for: Production of functional eggs and sperm from in vitro-expanded type A spermatogonia in rainbow trout
Source: Commun Biol. 2020 Jun 15;3:308. doi: 10.1038/s42003-020-1025-y (PMC7296041; doi:10.1038/s42003-020-1025-y)
Supplement: Supplementary file 5 — Reporting Summary [file 42003_2020_1025_MOESM5_ESM.pdf]

nature research

Corresponding author(s): Goro YOSHIZAKI
Last updated by author(s): May 10, 2020

Reporting Summary

Nature Research wishes to improve the reproducibility of the work that we publish. This form provides structure for consistency and transparency in reporting. For further information on Nature Research policies, see Authors & Referees and the Editorial Policy Checklist.

Statistics

For all statistical analyses, confirm that the following items are present in the figure legend, table legend, main text, or Methods section.

n/a	Confirmed	
☐	☒	The exact sample size (*n*) for each experimental group/condition, given as a discrete number and unit of measurement
☐	☒	A statement on whether measurements were taken from distinct samples or whether the same sample was measured repeatedly
☐	☒	The statistical test(s) used AND whether they are one- or two-sided<br>*Only common tests should be described solely by name; describe more complex techniques in the Methods section.*
☒	☐	A description of all covariates tested
☒	☐	A description of any assumptions or corrections, such as tests of normality and adjustment for multiple comparisons
☐	☒	A full description of the statistical parameters including central tendency (e.g. means) or other basic estimates (e.g. regression coefficient) AND variation (e.g. standard deviation) or associated estimates of uncertainty (e.g. confidence intervals)
☒	☐	For null hypothesis testing, the test statistic (e.g. *F*, *t*, *r*) with confidence intervals, effect sizes, degrees of freedom and *P* value noted<br>*Give P values as exact values whenever suitable.*
☒	☐	For Bayesian analysis, information on the choice of priors and Markov chain Monte Carlo settings
☒	☐	For hierarchical and complex designs, identification of the appropriate level for tests and full reporting of outcomes
☒	☐	Estimates of effect sizes (e.g. Cohen's *d*, Pearson's *r*), indicating how they were calculated

Our web collection on statistics for biologists contains articles on many of the points above.

Software and code

Policy information about availability of computer code

Data collection	All micrographs were taken using DP-73 (Olympus). Details of flowcytometry are mentioned below.
Data analysis	Prism7 for statistical analysis

For manuscripts utilizing custom algorithms or software that are central to the research but not yet described in published literature, software must be made available to editors/reviewers. We strongly encourage code deposition in a community repository (e.g. GitHub). See the Nature Research guidelines for submitting code & software for further information.

Data

Policy information about availability of data

All manuscripts must include a data availability statement. This statement should provide the following information, where applicable:

- Accession codes, unique identifiers, or web links for publicly available datasets
- A list of figures that have associated raw data
- A description of any restrictions on data availability

The data that support the findings of this study are available from the corresponding author upon reasonable request.
Also, all data sets are shown in sup data.

Field-specific reporting

Please select the one below that is the best fit for your research. If you are not sure, read the appropriate sections before making your selection.

☒ Life sciences ☐ Behavioural & social sciences ☐ Ecological, evolutionary & environmental sciences

For a reference copy of the document with all sections, see nature.com/documents/nr-reporting-summary-flat.pdf

Life sciences study design

All studies must disclose on these points even when the disclosure is negative.

Sample size	Sample sizes were determined on literature precedence for germ cell culture of other animals.
Data exclusions	No data were excluded from the analyses.
Replication	Culture experiments were performed in triplicate or more replicates. Replication of mating studies were restricted by the numbers of mature fish in each group. All replicate numbers of each mating were clearly described in the text.
Randomization	Cell cultures were performed under identical conditions. No randomization was used.
Blinding	Cell cultures were performed under identical conditions. No blinding was used.

Reporting for specific materials, systems and methods

We require information from authors about some types of materials, experimental systems and methods used in many studies. Here, indicate whether each material, system or method listed is relevant to your study. If you are not sure if a list item applies to your research, read the appropriate section before selecting a response.

Materials & experimental systems

n/a	Involved in the study	
☒	☐	Antibodies
☐	☒	Eukaryotic cell lines
☒	☐	Palaeontology
☐	☒	Animals and other organisms
☒	☐	Human research participants
☒	☐	Clinical data

Methods

n/a	Involved in the study	
☒	☐	ChIP-seq
☐	☒	Flow cytometry
☒	☐	MRI-based neuroimaging

Eukaryotic cell lines

Policy information about cell lines

Cell line source(s)	Sertoli cell line derived from rainbow trout testicular cells.
Authentication	None of the cell lines used were authentication.
Mycoplasma contamination	the cell lines were not tested for mycoplasma contamination.
Commonly misidentified lines (See ICLAC register)	none

Animals and other organisms

Policy information about studies involving animals; ARRIVE guidelines recommended for reporting animal research

Laboratory animals	Wild-type and albino rainbow trout were used for this study. All trouts were maintained in Oizumi station of TUMSAT for more than 30 years.
Wild animals	This study did not involve wild animals.
Field-collected samples	This study did not involve samples collected from the field.
Ethics oversight	IACUC of Tokyo University of Marine Science and Technology

Note that full information on the approval of the study protocol must also be provided in the manuscript.

Flow Cytometry

Plots

Confirm that:

☒ The axis labels state the marker and fluorochrome used (e.g. CD4-FITC).

☒ The axis scales are clearly visible. Include numbers along axes only for bottom left plot of group (a 'group' is an analysis of identical markers).

☒ All plots are contour plots with outliers or pseudocolor plots.

☐ A numerical value for number of cells or percentage (with statistics) is provided.

Methodology

Sample preparation	For rainbow trout Sertoli cell preparation, immature testes were dissociated with a trypsin solution containing 0.43 U/ ml trypsin (Worthington Biochemical Corporation, Lakewood, NJ, USA), 1 mM CaCl2, 5% fetal bovine serum (FBS, Thermo Fisher Scientific, Massachusetts, USA), and 40 U/ ml DNase I (Sigma-Aldrich, Missouri , USA) in PBS (pH 8.2) at 10°C for 2.5 hours. These dissociated cells including non-dissociated cell clumps were eliminated through a filtration of 42-µm-pore size nylon screen (NBC Industries, Tokyo, Japan). These cells were washed with H-MEM-5 (Hank's MEM supplemented with 25 mM HEPES and 5% FBS) and then isolated Sertoli cells cells by flow cytometer. For analysis of the number of cultured cells, cultured cells were detached from plate bottoms with 0.25% Trypsin-EDTA solution (0.25% Trypsin (BD Biosciences, New Jersey, USA) with 0.02% EDTA solution (Thermo Fisher Scientific)) at 10°C for 10 minutes. Then there cells were dissociated with a trypsin solution containing 0.43 U/ ml trypsin (Worthington Biochemical Corporation), 1 mM CaCl2, 5% fetal bovine serum (hermo Fisher Scientific), and 40 U/ ml DNase I (Sigma-Aldrich) in PBS (pH 8.2) at 10°C for over 20 minutes.
Instrument	MoFlo XDP, Cell Sorter (Beckman Coulter, California, USA) for isolation of Sertoli cells.<br>Guava easyCyte (Merck Millipore, Massachusetts, USA) for analysis of the cell number.
Software	Summit v5.4 (Beckman Coulter) in Moflo XDP.<br>Cytosoft 5.3 in GUAVA
Cell population abundance	Among the cells sorted using DsRed intensity from inhibin-DsRed trout, 98.6% were DeRed positive.
Gating strategy	According to forward and side scatter, single dissociated cells were gated in order to exclude dead and abnormal shape cells.<br>GFP and DsRed positive cells were counted according to green and red fluourescence intensity.

☒ Tick this box to confirm that a figure exemplifying the gating strategy is provided in the Supplementary Information.
